# Association between blood selenium levels and gestational diabetes mellitus: A systematic review and meta-analysis

**DOI:** 10.3389/fnut.2022.1008584

**Published:** 2022-11-23

**Authors:** Shuai Yan, Han Su, Yang Xia, Zixuan Yan, Yitao Gao, Mengyuan Shi, Huiyuan Liu, Yu Wen, Yuhong Zhao, Qing Chang

**Affiliations:** ^1^Liaoning Key Laboratory of Precision Medical Research on Major Chronic Disease, Shengjing Hospital of China Medical University, Shenyang, China; ^2^Department of Clinical Epidemiology, Shengjing Hospital of China Medical University, Shenyang, China; ^3^Department of Health Management, Shengjing Hospital of China Medical University, Shenyang, China

**Keywords:** gestational diabetes mellitus, selenium, association, meta-analysis, systematic review

## Abstract

**Introduction:**

The association between blood (serum or plasma) selenium concentrations and gestational diabetes mellitus (GDM) has been evaluated in some studies. However, the reported findings are debatable, and only case-control and cross-sectional studies were included.

**Objective:**

This research aimed to assess the association between blood selenium levels and GDM by analyzing existing literature. To provide a reference for the prevention and treatment of GDM, we included prospective studies which are not included in previous studies to collate more high-quality evidence and better test the etiological hypothesis between blood Se concentrations and GDM.

**Methods:**

The PubMed, EMBASE, and Web of Science databases were retrieved for literature up to September 2022, and relevant references were manually searched. Raw data from relevant studies were extracted, and a random effect model was adopted for meta-analysis. The total effects were reported as weighted mean differences. All data were analyzed using Stata 16.0 software.

**Results:**

Fourteen studies involving 890 pregnant women with GDM and 1618 healthy pregnant women were incorporated in the meta-analysis. Pregnancies with GDM had significantly lower blood selenium levels than those with normal glucose tolerance (weighted mean difference = −8.11; 95% confidence interval: −12.68 to −3.54, *P* = 0.001). Subgroup analyses showed that the association between blood selenium levels and GDM was consistent in the residents of Asia and Africa, but not in European. This trend was significant in the second and third trimester subgroups, but not in the first trimester subgroup. Articles published in 2006–2015 also showed this trend, but those published before 2005 and 2016–2019 did not show significant results. This difference was evident in non-prospective studies, but not significant in prospective studies. Studies using the Carpenter and Coustan diagnostic criteria were consistent with this trend, whereas studies using other diagnostic criteria found no differences. In addition, in terms of blood selenium measurement methods, atomic absorption spectrometry showed more significant differences than other methods. In the subgroup analysis based on the sample size of included studies and the quality of the studies, each subgroup showed statistical differences.

**Conclusion:**

Lower blood selenium concentrations are associated with GDM as shown in our study. Therefore, supplementing an appropriate amount of selenium may be helpful for GDM prevention and treatment.

## Introduction

Gestational diabetes mellitus (GDM) refers to diabetes that is first detected in women when they were pregnant. It is the most frequent pregnancy complication (1). As stated in the International Diabetes Federation Diabetes Atlas 10th edition, approximately 21.1 million (16.7%) women giving live births in 2021 experienced some form of hyperglycemia during pregnancy. The cause of hyperglycemia in 80.3% women was GDM. The prevalence of GDM in China, which was 8.6% in 2021, has been increasing in recent years. GDM often leads to poor postpartum outcomes, including polyhydramnios, pre-eclampsia, shoulder dystocia, birth canal tears, cesarean section, neonatal hypoglycemia, fetal overgrowth, and jaundice (2, 3). Additionally, women with a previous GDM are at a raised hazard of metabolic syndrome and renal, hepatic, cardiovascular, and retinal diseases (4, 5). Recent studies have suggested that certain nutrients, such as inositol, magnesium, zinc, selenium, vitamins D and B6, fatty acids, and probiotics, help prevent or treat GDM (6).

Selenium is an indispensable trace element that plays a pivotal role in human health. It is an important part of many enzymes and proteins in the body, including glutathione peroxidase, mitochondrial capsule selenoprotein, iodothyronine deiodinases, selenoprotein P, and selenoprotein W (7). Selenium has been linked to many diseases, including heart disease, autoimmune thyroid disease, diabetes in certain populations, and some types of cancers (8–10). The etiology, pathogenesis, and complications of diabetes are related to oxidative stress (7). Many researchers have studied the function of selenium in the prevention and treatment of diabetes because of its antioxidant effect (11, 12), reporting that selenium regulates glucose tolerance (13, 14).

Further, some studies have revealed that high blood selenium concentrations are related to a low diabetes prevalence (15, 16), while others have highlighted positive correlations between plasma selenium and fasting plasma glucose levels (17, 18). A growing number of studies on the association between selenium and diabetes have been conducted; however, controversial findings have been reported. Previous meta-analyses on the association between selenium levels and GDM by Askari (19), Kong (20), and Xu et al. (21) only included cross-sectional studies and case-control studies. Therefore, in our systematic review and meta-analysis, we searched the relevant literature and included other higher quality prospective studies (prospective cohort studies and nested case-control studies) to comprehensively analyze the association between maternal blood selenium levels and GDM and draw more reliable conclusions.

## Methods

The Meta-Analyses and Systematic Reviews of Observational Studies guidelines followed by our study (22). In addition, the Preferred Reporting Items for Systematic Reviews and Meta-Analyses (PRISMA) statement was observed to analyze data and report the results (23).

### Search strategy

We retrieved the EMBASE, PubMed, and Web of Science and extracted all relevant articles posted as of September 2022 using a combination of medical subject headings terms (“Diabetes, Gestational”and “Selenium”) and entree terms (“Pregnancy diabetes mellitus” and “selenium blood level”). The following free words were searched together with subject headings: “Diabetes, Pregnancy Induced,” “Pregnancy-Induced Diabetes,” “Diabetes, Pregnancy-Induced,” “Gestational Diabetes,” “Gestational Diabetes Mellitus,” “GDM,” “Diabetes Mellitus, Gestational,” “diabetic pregnancy,” “diabetes pregnancy,” “insulin gestation,” “selenate,” “selenium compounds,” “selenium supplement,” “selenium-binding proteins,” “plasma selenium,” “serum selenium,” and “blood selenium.” Relevant references of the extracted research were manually searched to identify potential studies for inclusion.

### Selection criteria

Studies compliant with the series of criteria were included: (1) the experimental group or case group of the study included patients with GDM and the control group of the study included healthy pregnant women, (2) all participants had no history of diabetes or related diseases of abnormal glucose tolerance before pregnancy, (3) blood selenium measurement values of patients diagnosed with GDM and healthy pregnant women were reported, and (4) full-text was written in English. Studies for which the full text and specific experimental results could not be obtained, animal experiments or *in vitro* experiments, and studies not reporting clear numerical values or not comparing patients with GDM with healthy pregnant women were excluded. Participants were required to have no prepregnancy diabetes or a family history of heart or kidney disease, hypertension, diabetes, and no recent use of Se supplements.

### Data extraction and quality assessment

All retrieved studies were reviewed independently by two reviewers, and they extracted data using pre-established forms. If there was a disagreement, it was discussed, and if no agreement could be reached, a third experienced researcher made the judgment. Data on the following was extracted from each research: first author, year of study completion, country of the study, type of study, samples and methods for determination of blood selenium levels, diagnostic criteria for GDM, number of participants, mean and standard deviation of blood selenium concentrations in the GDM and healthy pregnancies groups (except the values reported in Bo’s study, those reported in all other studies were converted to the same unit, μg/L. In the study by Zhu et al. (24), the results were expressed as medians and 25th–75th percentiles. When more data were not available, the methods used by Wan et al. (25), Luo et al. (26), and McGrath et al. (27) were used to convert the corresponding data into mean and standard deviation), trimester in which selenium levels were measured, average age, and body mass index. The Newcastle–Ottawa Scale (NOS), and the Agency for Healthcare Research and Quality (AHRQ) were used for quality assessment.

### Statistical analysis

Differences in blood selenium levels between groups were evaluated using weighted mean differences (WMDs) and 95% confidence intervals (95% CIs). *Z*-test was used to determine the significance levels. Forest plots were represented using the WMD and 95% CI for each study. Cochran’s Q statistic and *I*^2^ statistic were used to assess the heterogeneity of the included studies. Depending on the heterogeneity (*I*^2^ = 97.7%), the random effect model was used.

Subgroup analyses were performed to probe the potential sources of heterogeneity by stratifying different studies according to region, pregnancy period, study type, published period, GDM criteria, blood selenium measurement method, study quality, and sample size (It should be noted that in the pregnancy period subgroups, different studies were divided into first, second, and third trimester groups according to the pregnancy period when blood samples were collected. The current general pregnancy period was divided into the first (<13 weeks), second (14–27 weeks), and third (>28 weeks) trimesters. Most included studies were classified according to this standard. However, in Tan et al.’s (28) study, 20–33 and 33–42 weeks were considered as the second and third trimesters, and in Bo et al.’s (29) study 24–30 weeks was considered as the second trimester. In the study type subgroups, many types of studies were included, but the number of some types of studies was small. Therefore, nested case-control studies, prospective cohort studies, and randomized controlled trials were classified as prospective studies, and case-control and cross-sectional studies were classified as non-prospective studies).

Begg’s test and Egger’s test were utilized to assess potential publication bias. Sensitivity analyses were adopted by sequentially omitting each study to seek potential heterogeneity sources, and evaluate relevant changes in the pooled results.

All data were analyzed using Stata/SE 16.0 software (StataCorp LLC, TX, US). The significance level was set at 0.05, except in Cochran’s Q test for heterogeneity, in which it was set at 0.1.

## Results

**FIGURE 1:**
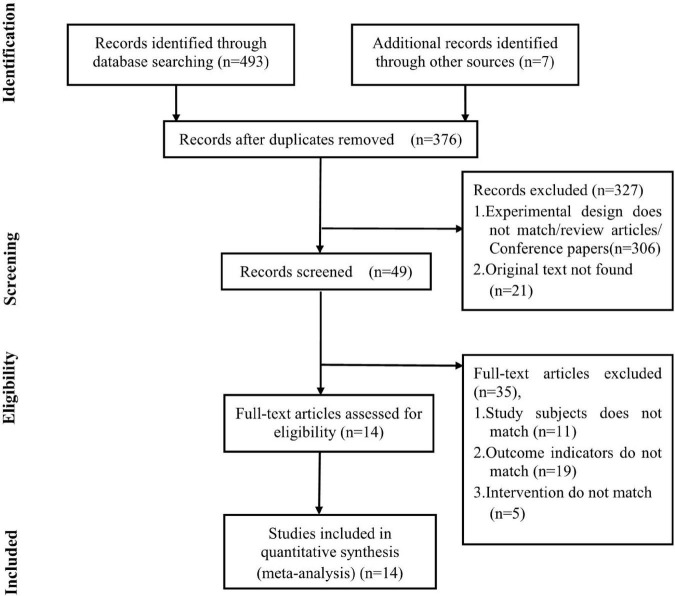
Flow diagram of study recruitment.

A total of 376 relevant articles were retrieved and screened by two independent reviewers. We excluded 327 studies (including articles whose experimental design did not match our study design, review articles, conference papers, and studies not reporting original data) by reading the titles and abstracts. Subsequently, 49 studies underwent detailed full-text review. Among them, 35 studies were excluded due to discrepancies in study subjects, intervention, and outcome indicators. Finally, data were fetched from 14 studies that conformed to the inclusion criteria (Figure 1).

### Study characteristics

Table 1 shows the main characteristics of the included studies, and additional details are presented in Supplementary Table 1. A total of 14 relevant studies published between 1984 and 2021 were included. These studies included a total of 890 women with GDM and 1618 healthy pregnancies (Moshfeghy et al. (30) measured the data during two different pregnancy periods in the same GDM and healthy pregnant women groups in one study). A total of 877 subjects participated in non-prospective studies [644 and 233 subjects in case-control studies (28, 29, 31–35) and cross-sectional studies (36–38), respectively] and 1631 subjects participated in prospective studies (685 and 946 subjects in nested case-control studies (24, 30), and prospective cohort studies (39, 40), respectively). Collected blood samples for selenium content measurement during the first (30, 38–40), second (24, 28–30, 33, 35, 36), and third (28, 31, 32, 34, 37) trimesters. The subjects were from Asia (24, 28, 30–32, 40), Europe (29, 33, 35–39), and Africa (34). Based on the mean age of the study subjects, most women were of the childbearing age (25–35 years). In all studies in which data on body mass index were available, the mean body mass index of the subjects was < 28 kg/m^2^, except in the research by Al-Saleh et al. (32), which included pregnant women with an average body mass index of ≥28 kg/m^2^. Most studies used atomic absorption spectrometry (AAS) to measure blood selenium levels (29–34, 36, 38). Tan et al. (28) used atomic fluorescence spectrometry (AFS); Hyvönen-Dabek et al. (37) used proton-induced X-ray emission (PIXE);and Lewandowska et al. (39), Onat et al. (35), Zhu et al. (24), and Liu et al. (40) used inductively coupled plasma mass spectrometry (ICP-MS) to measure blood selenium levels. Bo et al. (29), Kilinc et al. (36), Hamdan et al. (34), Moshfeghy et al. (30), and Onat et al. (35) adopted the Carpenter and Coustan (C&C) diagnostic criteria; Al-Saleh et al. (32) and Molnar et al. (33) adopted the World Health Organization (WHO) diagnostic criteria; Lewandowska et al. (39), Zhu et al. (24), and Liu et al. (40) adopted the International Association of Diabetes and Pregnancy Study Group criteria (IADPSG); and Eroğlu et al. (38) adopted the American College of Obstetricians and Gynecologists criteria (ACOG).

**TABLE 1:** Characteristics of studies included in the meta-analysis^a^.

Study*	Location	Study type	GDM	HPW	Blood Se (μg/L)		Selenium measurement trimester
					
			(*n*)	(*n*)	GDM	NPW	
Hyvönen-Dabek (37)	Finland	Case-control	5	10	17 ± 8	28 ± 10	Third
Tan et al. (28)	China	Case-control	57	40	66.0 ± 12.0	78.5 ± 17.7	Second
		Case-control	83	50	61.5 ± 13.1	70.7 ± 15.2	Third
Al-Saleh (31)	Kuwait	Case-control	15	15	75.2 ± 3.1	102.3 ± 3.1	Third
Bo (29)	Italy	Case-control	29	123	8.8 ± 1.3**	10.8 ± 1.8**	Second
Al-Saleh et al. (32)	Kuwait	Case-control	10	11	85.1 ± 5.4	89 ± 4.9	Third
Kilinc (36)	Turkey	Cross-sectional	30	101	34.7 ± 8.7	50.7 ± 9.8	Second
Molnar (33)	Hungary	Case-control	17	20	51.7 ± 11.6	40.5 ± 8.0	Second
Hamdan (34)	Sudan	Case-control	31	31	164.4 ± 59.0	204 ± 78.8	Third
Lewandowska (39)	Poland	Prospective cohort	110	453	61.88 ± 44.39	60.48 ± 40.91	First
Moshfeghy (30)	Iran	Nested case-control	25	50	50.60 ± 10.88	66.02 ± 10.57	First
					39.87 ± 10.23	63.17 ± 10.22	Second
Eroğlu (38)	Turkey	Cross-sectional	43	44	55.00 ± 8.07	53.08 ± 7.47	First
Onat (35)	Turkey	Case-control	60	52	29.48 ± 9.87	38.21 ± 11.56	Second
Liu (40)	China	Prospective cohort	70	313	69.0 ± 15.0	66.3 ± 12.9	First
Zhu (24)	China	Nested case-control	305	305	29.48 ± 7.78	31.03 ± 8.01	Second

^a^GDM, gestational diabetes mellitus; HPW, healthy pregnant women; RCT, randomized controlled trial.

*First author (year of study completion).

**μmol/L.

Begg’s test (*P* = 0.620) and Egger’s test (*P* = 0.152) indicated that there was no statistical publication bias (Supplementary Figure 1). Sensitivity analysis showed that no study significantly deviated from other studies (Supplementary Figure 2). Supplementary Table 2 shows the quality assessment of the included studies. Based on the quality assessment by the NOS, the average value for the 8 case-control and 4 cohort studies was 6.5 stars. All included studies scored between 5 and 7, we consider these studies to be of medium or high-quality because the criteria for study quality is still inadequate. We used The AHRQ scale for the evaluation of the cross-sectional study, including 11 items. Each item was rated as “yes” (1 point), and “no” or “unclear” (0 point); 0–3 points represented low-quality literature, 4–7 points represented medium-quality literature, and 8–11 points represented high-quality literature. Both studies were of medium-quality with scores of 6–7.

### The association between blood Se concentration and gestational diabetes mellitus

**FIGURE 2:**
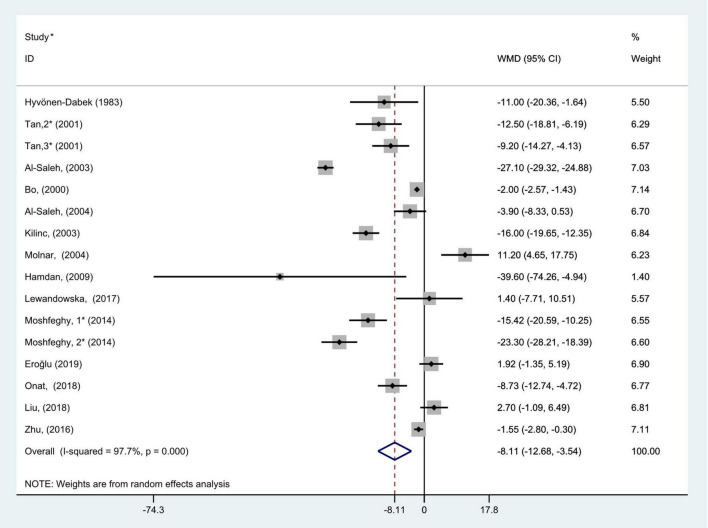
Forest plot of the blood selenium level in patients with gestational diabetes mellitus and healthy pregnant women. The random effects model is applied. For studies in which blood selenium levels were measured during different pregnancy periods in one study, 1, 2, and 3 are used to denote measurements performed in the first, second, and third trimesters, respectively. *First author (year of study completion).

The general level of blood (serum or plasma) selenium concentration in gravidas with GDM was significantly less than that in healthy pregnancies with normal glucose tolerance (WMD = −8.11; 95% CI: −12.68 to −3.54, *P* = 0.001, Figure 2). Because the heterogeneity was considered significant (*P* < 0.001, *I*^2^ = 97.7%), we used the random effects model to analyze the WMDs from individual studies.

### Subgroup analysis

The comprehensive results of subgroup analyses are shown in Figures 3–10.

**FIGURE 3:**
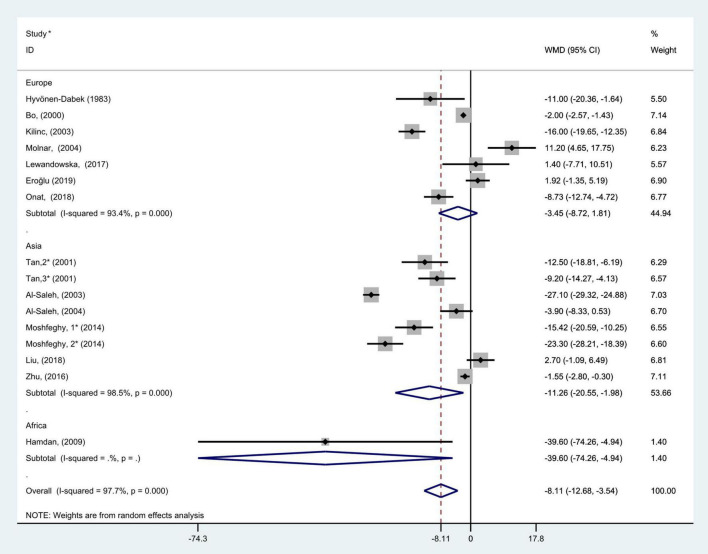
Subgroup analysis of blood selenium level in patients with gestational diabetes mellitus and healthy pregnant women based on geographic region. The random effects model is applied. For studies in which blood selenium levels were measured during different pregnancy periods in one study, 1, 2, and 3 are used to denote measurements performed in the first, second, and third trimesters, respectively. *First author (year of study completion).

**FIGURE 4:**
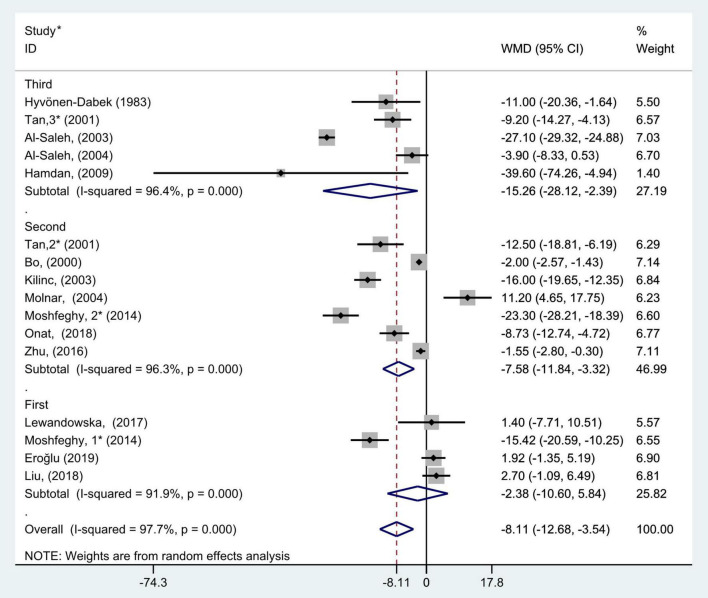
Subgroup analysis of blood selenium level in patients with gestational diabetes mellitus and healthy pregnant women based on different trimesters. The random effects model is applied. For studies in which blood selenium levels were measured during different pregnancy periods in one study, 1, 2, and 3 are used to denote measurements performed in the first, second, and third trimesters, respectively. *First author (year of study completion).

**FIGURE 5:**
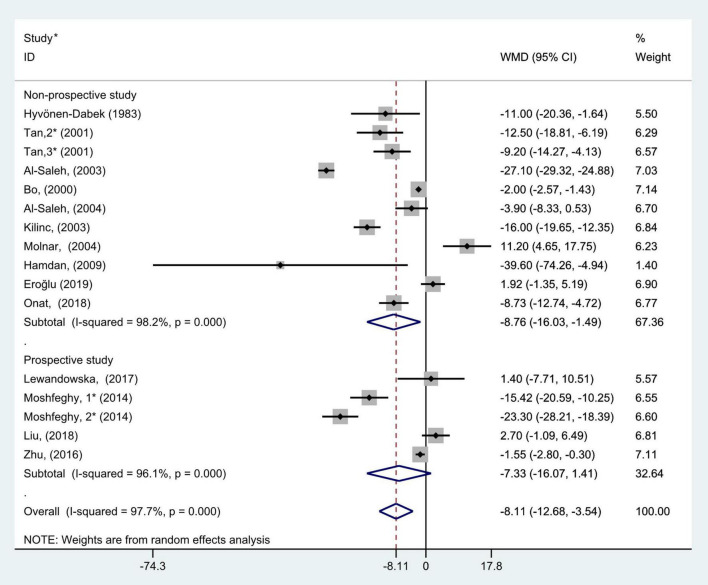
Subgroup analysis of blood selenium level in patients with gestational diabetes mellitus and healthy pregnant women based on study types. The random effects model is applied. For studies in which blood selenium levels were measured during different pregnancy periods in one study, 1, 2, and 3 are used to denote measurements performed in the first, second, and third trimesters, respectively. *First author (year of study completion).

**FIGURE 6:**
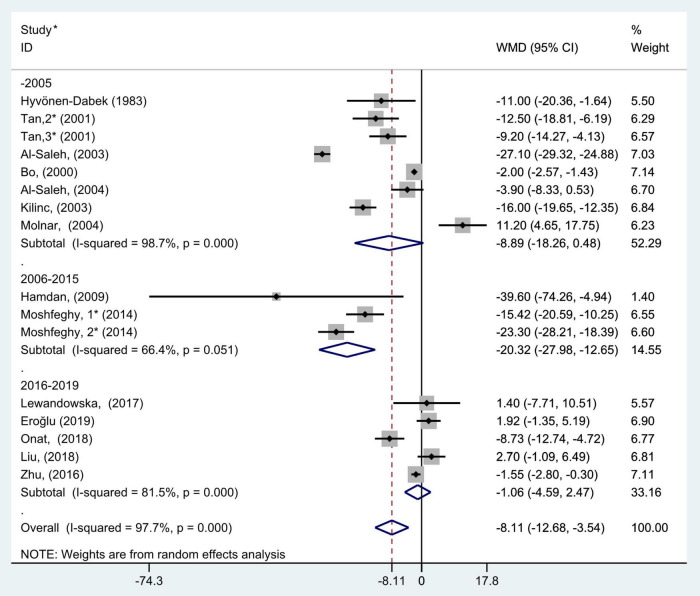
Subgroup analysis of blood selenium level in patients with gestational diabetes mellitus and healthy pregnant women based on study completion period. The random effect model is applied. For studies in which blood selenium levels were measured during different pregnancy periods in one study, 1, 2, and 3 are used to denote measurements performed in the first, second, and third trimesters, respectively. *First author (year of study completion).

**FIGURE 7:**
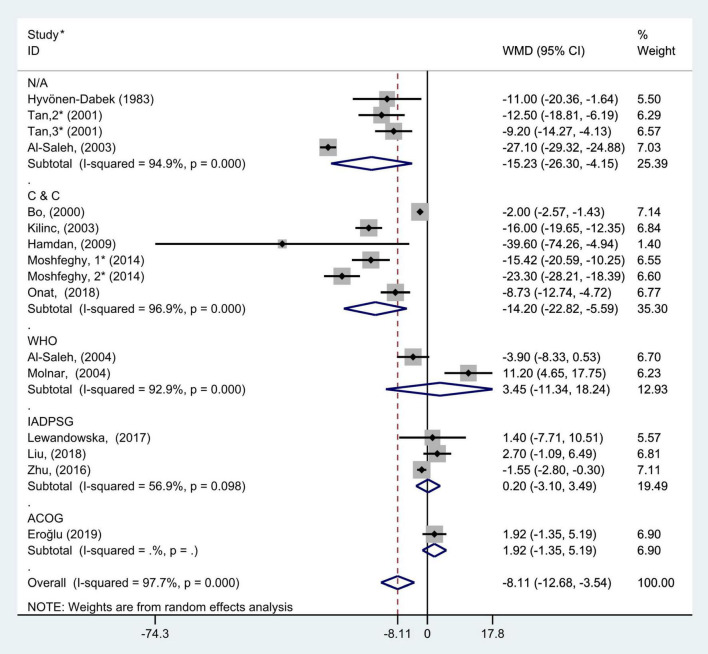
Subgroup analysis of blood selenium level in patients with gestational diabetes mellitus and healthy pregnant women based on different GDM criteria. The random effect model is applied. N/A, not available or not reported; C&C, carpenter and coustan; WHO, World Health Organization; IADPSG, International Association of Diabetes and Pregnancy Study Groups; ACOG, American College of Obstetricians and Gynecologists. For studies in which blood selenium levels were measured during different pregnancy periods in one study, 1, 2, and 3 are used to denote measurements performed in the first, second, and third trimesters, respectively. *First author (year of study completion).

**FIGURE 8:**
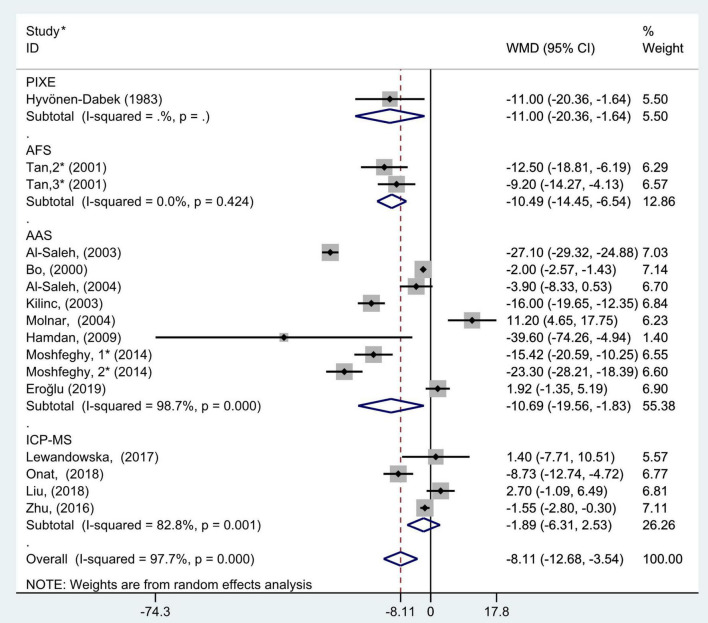
Subgroup analysis of blood selenium level in patients with gestational diabetes mellitus and healthy pregnant women based on blood selenium measurement methods. The random effect model is applied. PIXE, proton-induced x-ray emission; AFS, atomic fluorescence spectrometric; AAS, atomic absorption spectrometry; ICP-MS, inductively coupled plasma mass spectrometry. For studies in which blood selenium levels were measured during different pregnancy periods in one study, 1, 2, and 3 are used to denote measurements performed in the first, second, and third trimesters, respectively. *First author (year of study completion).

**FIGURE 9:**
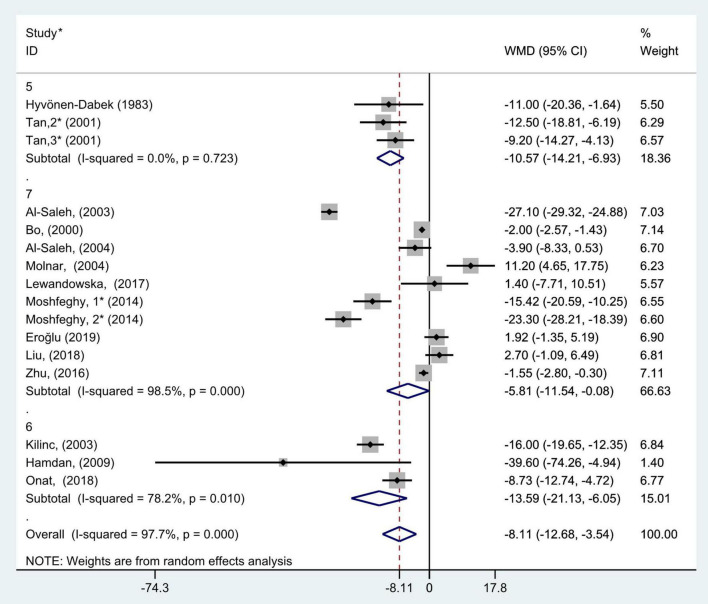
Subgroup analysis of blood selenium level in patients with gestational diabetes mellitus and healthy pregnant women based on the score of study quality. The random effect model is applied. For studies in which blood selenium levels were measured during different pregnancy periods in one study, 1, 2, and 3 are used to denote measurements performed in the first, second, and third trimesters, respectively. *First author (year of study completion).

**FIGURE 10:**
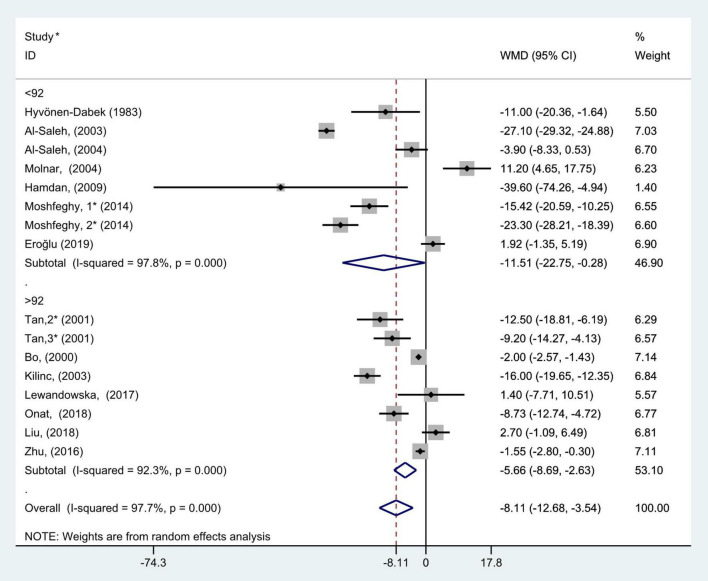
Subgroup analysis of blood selenium level in patients with gestational diabetes mellitus and healthy pregnant women based on sample size. The random effect model is applied. For studies in which blood selenium levels were measured during different pregnancy periods in one study, 1, 2, and 3 are used to denote measurements performed in the first, second, and third trimesters, respectively. *First author (year of study completion).

Subgroup analysis according to geographic location showed that there was considerable heterogeneity in the Asian and Europen studies (*P* < 0.001, *I*^2^ = 98.5%; *P* < 0.001, *I*^2^ = 93.4%, respectively). Pooled analysis was operated using the random effects model. The results showed that blood selenium concentrations in patients with GDM were noticeably less than those in healthy pregnancies with normal glucose tolerance in Asia and Africa (WMD = −11.26, 95% CI: −20.55 to −1.98, *P* = 0.02 and WMD = −39.60, 95% CI: −74.26 to −4.94, *P* = 0.03, respectively). However, there was no statistical difference in blood selenium contents between patients with GDM and healthy pregnancies in Europe (WMD = −3.45; 95% CI: −8.72 to 1.81, *P* = 0.20, Figure 3).

The results of pregnancy period subgroup analysis showed that there was great heterogeneity in the studies according to each pregnancy period (first trimester: *P* < 0.001, *I*^2^ = 91.9%; second trimester: *P* < 0.001, *I*^2^ = 96.3%; and third trimester: *P* < 0.001, *I*^2^ = 96.4%). Pooled analysis was operated using the random effects model. The results also indicated that the blood selenium levels in patients with GDM were significantly less than that of healthy pregnancies in the second and third trimester groups (WMD = −7.58, 95% CI: −11.84 to −3.32, *P* < 0.001 and WMD = −15.26, 95% CI: −28.12 to −2.39, *P* = 0.02, respectively), while this was not observed in the first trimester group (WMD = −2.38; 95% CI: −10.60 to 5.84, *P* = 0.57, Figure 4).

To further probe the source of heterogeneity, subgroup analysis was also conducted on the study types. In the subgroup analysis grouped by study type, selecting the random effect model for pooled analysis because each group had marked heterogeneity (non-prospective studies: *P* < 0.001, *I*^2^ = 98.2% and prospective studies: *P* < 0.001, *I*^2^ = 96.1%). The results of subgroup analysis demonstrated that blood selenium contents in gravidas with GDM were notably lower than those in healthy pregnancies with normal glucose tolerance in non-prospective studies (WMD = −8.76, 95% CI: −16.03 to −1.49, *P* = 0.02), but this difference was not significant in prospective studies (WMD = −7.33; 95% CI: −16.07 to 1.41, *P* = 0.10, Figure 5).

The results of subgroup analysis according to year of study completion (before 2006, 2006–2015, and 2016–2019) showed that there was large heterogeneity in the studies of each group (*P* < 0.001, *I*^2^ = 98.7%; *P* = 0.05, *I*^2^ = 66.4%; and *P* < 0.001, *I*^2^ = 81.5%, respectively). The random effects model was selected for pooled analysis. The results also indicated that blood selenium concentrations in women with GDM were noticeably less than those in pregnancies with normal glucose tolerance in the 2006–2015 group (WMD = −20.32, 95% CI: −27.98 to −12.65, *P* < 0.001), but this trend was not found in the before 2005 and 2016–2019 groups (WMD = −8.89, 95% CI: −18.26 to 0.48, *P* = 0.06 and WMD = −1.06, 95% CI: −4.59 to 2.47, *P* = 0.56, respectively, Figure 6).

The subgroup analysis based on the diagnostic criteria for GDM used in the included studies (the studies that did not report the diagnostic criteria were divided into N/A group) showed that there was considerable heterogeneity in the studies with different diagnostic criteria (C&C: *P* < 0.001, *I*^2^ = 96.9%; WHO: *P* < 0.001, *I*^2^ = 92.9%; IADPSG: *P* = 0.10, *I*^2^ = 56.9%; and N/A: *P* < 0.001, *I*^2^ = 94.9%). The random effect model was used for pooled analysis. The results showed that in C&C and N/A groups, the blood selenium concentration of gravidas with GDM was significantly lower than that of healthy pregnant women with normal glucose tolerance (WMD = −14.20, 95% CI: −22.82 to −5.59, *P* = 0.001 and WMD = −15.23, 95% CI: −26.30 to −4.15, *P* = 0.007, respectively). However, in the WHO, IADPSG, and ACOG groups, there was no statistical difference in blood selenium content between GDM patients and healthy pregnant women (WMD = 3.45, 95% CI: −11.34 to 18.24, *P* = 0.65; WMD = 0.20, 95% CI: −3.10 to 3.49, *P* = 0.91; and WMD = 1.92, 95% CI: −1.35 to 5.19, *P* = 0.25, respectively, Figure 7).

The subgroup analysis results of blood selenium measurement methods showed that there was great heterogeneity in the studies of different measurement methods (AAS: *P* < 0.001, *I*^2^ = 98.7%; ICP-MS: *P* = 0.001, *I*^2^ = 82.8%; and AFS: *P* = 0.42, *I*^2^ = 0.0%). Pooled analysis was conducted using the random effects model. The results also showed that the blood selenium level of women with GDM in AAS, AFS, and PIXE groups was significantly lower than that of healthy pregnant women (WMD = −10.69, 95% CI: −19.56 to −1.83, *P* = 0.02; WMD = −10.49, 95% CI: −14.45 to −6.54, *P* < 0.001; WMD = −11.00, 95% CI: −20.36 to −1.64, *P* = 0.02, respectively). However, this was not observed in ICP-MS group (WMD = −1.89; 95% CI: −6.31 to 2.53, *P* = 0.40, Figure 8).

According to the results of study quality assessment, the included studies were divided into 3 subgroups, 5, 6, and 7, and subgroup analysis was performed. Random effect model was selected because 6 and 7 points subgroups had marked heterogeneity (5 points: *P* = 0.72, *I*^2^ = 0.0%; 6 points: *P* = 0.01, *I*^2^ = 78.2%; and 7 points: *P* < 0.001, *I*^2^ = 98.5%). The results of subgroup analysis demonstrated that blood selenium contents in gravidas with GDM were notably lower than those in healthy pregnancies with normal glucose tolerance in all 5, 6, and 7 points subgroups (WMD = −10.57, 95% CI: −14.21 to −6.93, *P* < 0.001; WMD = −13.59, 95% CI: −21.13 to −6.05, *P* < 0.001; and WMD = −5.81, 95% CI: −11.54 to −0.08, *P* = 0.05, respectively, Figure 9).

According to the median of the sample size, all included studies were divided into two subgroups, >92 and <92. The results of subgroup analysis showed that there was large heterogeneity in the studies of each group (>92: *P* < 0.001, *I*^2^ = 92.3%; and <92: *P* < 0.001, *I*^2^ = 97.8%). The results of subgroup analysis demonstrated that blood selenium contents in gravidas with GDM were notably lower than those in healthy pregnancies with normal glucose tolerance in both >92 and <92 studies (WMD = −5.66, 95% CI: −8.69 to −2.63, *P* < 0.001 and WMD = −11.51, 95% CI: −22.75 to −0.28, *P* = 0.05, respectively, Figure 10).

Due to lack of sufficient study data and limitations of study design, subgroup analyses according to subjects’ mean age and body mass index were not performed.

## Discussion

Our study showed that blood selenium contents in pregnancies with GDM were notably lower than those in normal glucose tolerance pregnancies, especially in the Asian, African, second and third trimesters, studies completed in 2006–2015, used C&C as GDM criteria, and blood selenium was measured by AAS subgroups.

Compared with the previous research, we have the following innovations: Firstly, our research has developed clearer inclusion and exclusion criteria. Different from the research of Xu et al. (21), we exclude some studies that do not meet the inclusion criteria, and the results are more convincing. For example, in the paper published by Al-Saleh et al. (41) in 2005, the subjects of their study were women with insulin-dependent diabetes before pregnancy, not pregnant women with GDM, so this study was not included in our research. We set clearer inclusion and exclusion criteria to focus our included studies on GDM. Secondly, in order to incorporate more research data to draw more comprehensive conclusions, we have included more studies of different types, including prospective cohort studies and nested case-control studies that were not included in previous meta-analyses of Askari et al. (19) and Kong et al. (20). Our study included 2 prospective cohort studies (39, 40) and 2 nested case-control studies (24, 30), Among them, the papers of Lewandowska et al. (39), Moshfeghy et al. (30), and Liu et al. (40) were included in Xu et al.’s study as a cross-sectional study or a case-control study. We have made amendments to this and made further analysis according to the type of study. In addition, we conducted subgroup analyses from different perspectives not involved in previous studies (19–21), such as study type, study completion time, GDM diagnostic criteria, blood selenium measurement methods, quality of included studies, and study sample size, to further explore the source of heterogeneity.

In European studies, there was no considerable difference in blood selenium concentrations between pregnancies with GDM and those with normal glucose tolerance. It may be that Europeans consume meat, wheat, and milk, which are excellent sources of selenium, as their staple food and their eating habits may not be significantly altered during pregnancy. The diet of Asians and Africans generally includes grains and vegetables, which cannot supply adequate selenium. During pregnancy, insulin resistance in pregnancies with GDM leads to an increase in selenium demand; therefore, their blood selenium level is significantly different from that of healthy pregnancies.

The results of trimester subgroup analysis showed that blood selenium concentrations in pregnancies with GDM were significantly less than those in normal glucose tolerance pregnancies in the second and third trimesters; however, no significant difference was seen in the first trimester. This may due to the fact that the selenium demand of mothers and fetuses in the second and third trimesters is greater than that in the first trimester.

The results of subgroup analysis according to the research method demonstrated that the blood selenium contents of women with GDM in the non-prospective studies were significantly lower than that of healthy pregnant women. However, prospective studies did not show statistical differences. This may be caused by the prospective subgroup included studies by Lewandowska et al. (39) and Liu et al. (40), whose subjects were all pregnant women in the first trimester. Our subgroup analysis on trimester showed that there was no significant difference in blood selenium levels between GDM pregnant women and normal pregnant women in the first trimester, and the data of these two studies may have influenced the pooled results.

In addition, we found that the blood selenium concentration of pregnant women with GDM was significantly lower than that of pregnant women with normal glucose tolerance in the studies using C&C criteria, while no significant difference was found in the studies using other diagnostic criteria. This may be because the number of studies that used other criteria was relatively small, and the result is quite accidental.

In the study using AAS to measure the blood selenium level, we found that the blood selenium concentration of pregnant women with GDM was significantly lower than that of pregnant women with normal glucose tolerance. Due to the small number of studies using AFS and PIXE, the results may have certain contingencies. However, no significant difference was found in the study using ICP-MS. This may be caused by the ICP-MS subgroup included studies by Lewandowska et al. (39) and Liu et al. (40), whose subjects were all pregnant women in the first trimester. This may have had an impact on the pooled results.

The possible reasons for higher blood selenium contents in pregnant women with normal glucose tolerance than in patients with GDM are as follows: selenium can regulate energy metabolism at multiple levels, the antioxidant effect of selenium reduces the intensity of oxidative stress, and the intensity of oxidative stress can enhance insulin resistance and regulation of pancreas by selenium to a certain extent via β cell activity (42–45). In conclusion, pregnant women with low blood selenium levels are more likely to develop GDM. The study by Molnar et al. (33), Lewandowska et al. (39), Liu et al. (40), and Eroğlu et al. (38) yielded the opposite result, which might be due to underrepresentation of subjects recruited in the trial, differences in selenium content in the regions from which patients with GDM and controls were enrolled, measurement errors, and racial differences. The above reasons may also lead to large differences in blood selenium levels in different studies.

This study helps provide a new reference for the prevention and treatment of GDM, and the higher quality studies included in our meta-analysis help draw more convincing results than previous studies. However, our study has the following limitations: the measurement methods and blood samples for selenium measurement in different studies contained in this meta-analysis are different, diagnostic criteria of GDM used in different studies are different, original data of the study cannot be obtained, and accuracy of the study cannot be guaranteed. This might affect our study results. Some included studies did not provide more comprehensive baseline information; therefore, further analysis could not be conducted to explore the heterogeneity source. In addition, the number of prospective studies included is small. Differences in the dietary habits of the surveyed populations in different regions could also affect our study results. Therefore, our study findings should be interpreted with caution. More types of studies from different regions and including different races are needed to further prove the association between blood selenium levels and GDM.

In conclusion, the overall blood selenium content in patients with GDM was significantly lower than that in pregnant women with normal glucose tolerance, especially in the Asian, African, second and third trimesters, studies completed in 2006–2015, adopted C&C criteria, and measurement of blood selenium by AAS subgroups. Supplementation of selenium in women of childbearing age may be helpful for the prevention and treatment of GDM.

## Data availability statement

The original contributions presented in this study are included in the article/Supplementary material, further inquiries can be directed to the corresponding authors.

## Author contributions

SY and QC designed the study and formulated the clinical question. SY had full access to all data in the study and is responsible for data integrity and the accuracy of data analysis. SY and HS cleaned the data, checked the discrepancy, and analyzed the data. HS, YX, ZY, YG, MS, HL, YW, and YZ: writing – review and editing. QC: conceptualization, resources, writing – review and editing, supervision, and funding acquisition. All authors collected, managed, analyzed the data, prepared, reviewed, revised, and read and approved the final manuscript.
