# Supplementary material for: Association between blood selenium levels and gestational diabetes mellitus: A systematic review and meta-analysis
Source: Front Nutr. 2022 Nov 23;9:1008584. doi: 10.3389/fnut.2022.1008584 (PMC9726795; doi:10.3389/fnut.2022.1008584)
Supplement: Supplementary file 1 [file Data_Sheet_1.docx]

**Supplementary Material**

## The association between blood selenium level and gestational diabetes mellitus：A systematic review and meta-analysis

Catalog

**Supplemental Table 11**

**Supplemental Figure 12**

**Supplemental Figure 23**

**Supplemental Table 24**

Case-Control Studies4

Cohort Studies4

Cross-sectional Studies4

**Supplemental Table 1: Basic information on included studies**^a^

|  |  |  |  |  |  |  |  |  |  |  |  | Selenium  measurement  trimester |  |  |
| --- | --- | --- | --- | --- | --- | --- | --- | --- | --- | --- | --- | --- | --- | --- |
| Study^b^ | Location | Study type | Sample | Methods | GDM | GDM | HPW | Gestational age (week) | | Blood Se(μg/L) | |  | Average | Average |
|  |  |  |  |  | criteria | (n) | (n) | GDM | HPW | GDM | HPW |  | age | BMI (kg/m2) |
| Hyvönen-Dabek,1983 | Finland | Case-control | Serum | PIXE | N/A | 5 | 10 | N/A | N/A | 17 ± 8 | 28 ± 10 | Third | N/A | N/A |
| Tan,2001 | China | Case-control | Serum | AFS | N/A | 57 | 40 | 20-33 | 20-33 | 66.0 ± 12.0 | 78.5 ± 17.7 | Second | N/A | N/A |
|  |  | Case-control | Serum | AFS | N/A | 83 | 50 | 33-42 | 33-42 | 61.5 ± 13.1 | 70.7 ± 15.2 | Third | N/A | N/A |
| Al-Saleh,2003 | Kuwait | Case-control | Serum | AAS | N/A | 15 | 15 | 39 ± 0.3 | 40 ± 0.4 | 75.2 ± 3.1 | 102.3 ± 3.1 | Third | 31 | 28.57 |
| Bo,2000 | Italy | Case-control | Serum | AAS | C & C | 29 | 123 | 24–30 | 24-30 | 8.8 ± 1.3* | 10.8 ± 1.8* | Second | 33.5 | 25.4 |
| Al-Saleh,2004 | Kuwait | Case-control | Serum | AAS | WHO | 10 | 11 | 38 ± 0.4 | 40 ± 0.5 | 85.1 ± 5.4 | 89 ± 4.9 | Third | 32 | 36.26 |
| Kilinc,2003 | Turkey | Cross-sectional | Serum | AAS | C & C | 30 | 101 | 24-28 | 24–28 | 34.7 ± 8.7 | 50.7 ± 9.8 | Second | 25 | N/A |
| Molnar,2004 | Hungary | Case-control | Serum | AAS | WHO | 17 | 20 | 24-28 | 24–28 | 51.7 ± 11.6 | 40.5 ± 8.0 | Second | 31 | 24.1 |
| Hamdan,2009 | Sudan | Case-control | Serum | AAS | C & C | 31 | 31 | 33.2 ± 5.3 | 35.3 ± 4.4 | 164.4 ± 59.0 | 204 ± 78.8 | Third | 32 | 25.8 |
| Lewandowska,2017 | Poland | Prospective cohort | Serum | MS | IADPSG | 110 | 453 | 10-14 | 10-14 | 61.88 ± 44.39 | 60.48 ± 40.91 | First | 34.8 | 24.9 |
| Moshfeghy,2014 | Iran | nested case-control | Serum | AAS | C & C | 25 | 50 | 11-13 | 11-13 | 50.60 ± 10.88 | 66.02 ± 10.57 | First | 25.76 | 25.36 |
|  |  |  |  | AAS | C & C |  |  | 24-28 | 24-28 | 39.87 ± 10.23 | 63.17 ± 10.22 | Second |  |  |
| Eroğlu,2019 | Turkey | Cross-sectional | Serum | AAS | ACOG | 43 | 44 | 12.16 ± 0.75 | 12.22 ± 0.74 | 55.00 ± 8.07 | 53.08 ± 7.47 | First | 26.45 | 24.46 |
| Onat,2018 | Turkey | Case-control | Serum | ICP-MS | C & C | 60 | 52 | 24-28 | 24-28 | 29.48 ± 9.87 | 38.21 ± 11.56 | Second | 32.5 | 26.38 |
| Liu,2018 | China | Prospective cohort | Serum | ICP-MS | IADPSG | 70 | 313 | 7–11 | 7–11 | 69.0 ± 15.0 | 66.3 ± 12.9 | First | 29.2 | 22.8 |
| Zhu,2016 | China | nested case-control | plasma | ICP-MS | IADPSG | 305 | 305 | 24–28 | 24–28 | 29.48 ± 7.78 | 31.03 ± 8.01 | Second | 30.57 | N/A |

^a^ GDM, gestational diabetes mellitus; HPW, healthy pregnant women; PIXE, proton‐induced x‐ray emission; N/A, not available or not reported; AFS, atomic fluorescence spectrometry; AAS, atomic absorption spectrometry; C&C, carpenter and coustan; WHO, World Health Organization; MS, mass spectrometry; IADPSG, International Association of Diabetes and Pregnancy Study Groups; ACOG, American College of Obstetricians and Gynecologists; ICP-MS, inductively coupled plasma mass spectrometry.

^b^ First author (year of study completion)

* μmol/L.

**Supplemental Figure 1: Egger’s funnel plot of included studies for potential publication bias**


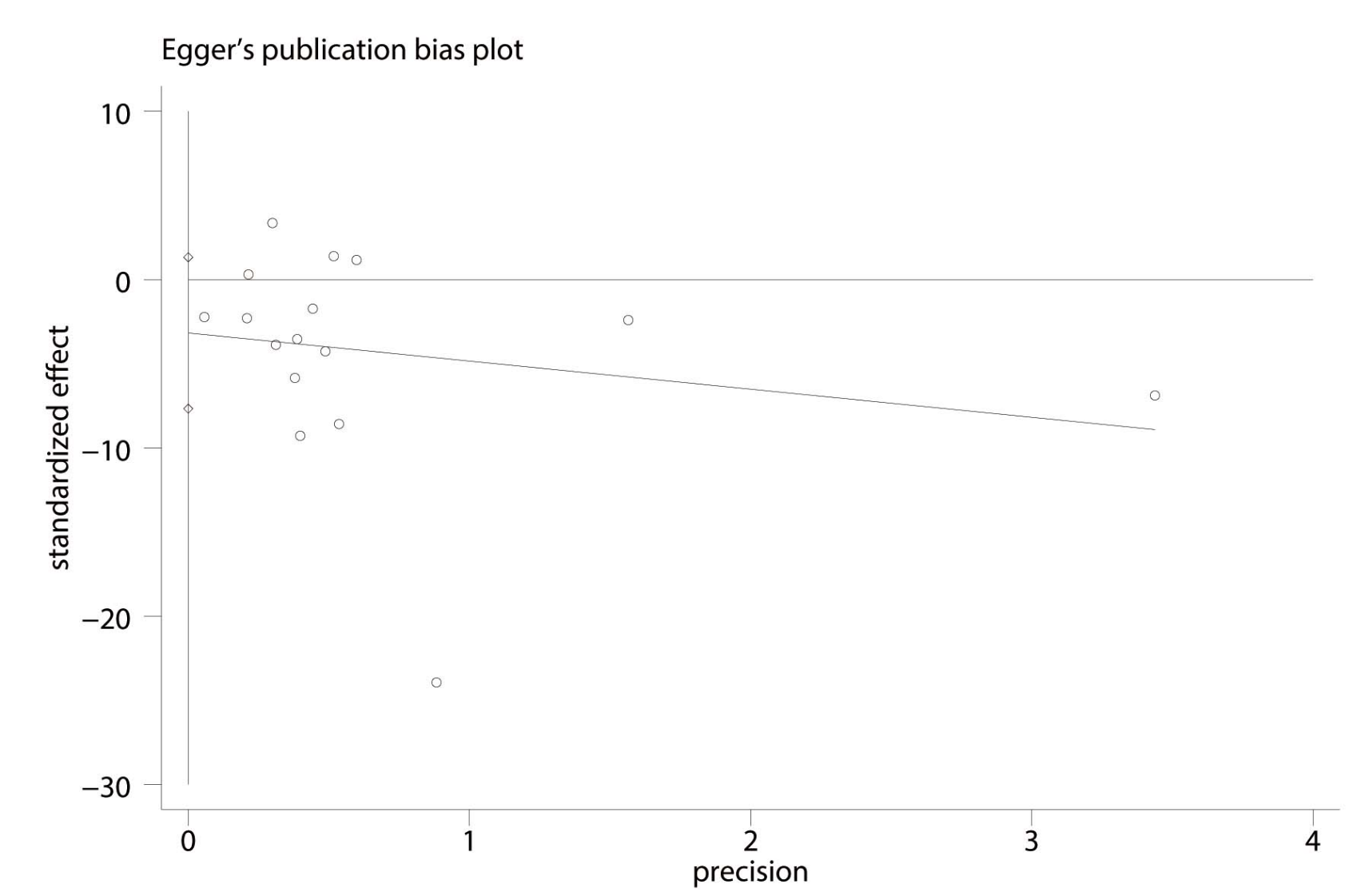


**Supplemental Figure 2:Sensitivity analysis of included studies^a^**





For studies in which blood selenium levels were measured during different pregnancy periods in one study, 1, 2, and 3 are used to denote measurements performed in the first, second, and third trimesters, respectively.

^a^ First author (year of study completion)

**Supplemental Table 2: Assessment of the quality of the included studies**

Case-Control Studies

| Study | Published year | Selection | Comparability | Exposure | Total |
| --- | --- | --- | --- | --- | --- |
| Hyvönen-Dabek | 1984 | ** | * | ** | 5 |
| Tan. | 2001 | ** | * | ** | 5 |
| Al-Saleh. | 2004 | *** | ** | ** | 7 |
| Bo. | 2005 | ** | ** | *** | 7 |
| Al-Saleh. | 2007 | *** | ** | ** | 7 |
| Molnar. | 2008 | *** | ** | ** | 7 |
| Hamdan. | 2014 | ** | ** | ** | 6 |
| Onat. | 2021 | ** | ** | ** | 6 |

Cohort Studies (Including prospective cohort studies and nested case-control studies)

| Study | Published year | Selection | Comparability | Outcome | Total |
| --- | --- | --- | --- | --- | --- |
| Lewandowska. | 2020 | **** | * | ** | 7 |
| Moshfeghy. | 2020 | *** | ** | ** | 7 |
| Liu. | 2021 | *** | * | *** | 7 |
| Zhu. | 2021 | **** | * | ** | 7 |

| Cross-sectional Studies |  |  |
| --- | --- | --- |
| **Item** | **Kilinc 2008** | **Eroğlu 2021** |
| 1) Define the source of information (survey, record review) | Yes | Yes |
| 2) List inclusion and exclusion criteria for exposed and unexposed subjects (cases and controls) or refer to previous publications | Yes | Yes |
| 3) Indicate time period used for identifying patients | Yes | Yes |
| 4) Indicate whether or not subjects were consecutive if not population-based | Yes | Yes |
| 5) Indicate if evaluators of subjective components of study were masked to other aspects of the status of the participants | Unclear | Unclear |
| 6) Describe any assessments undertaken for quality assurance purposes (e.g., test/retest of primary outcome measurements) | Yes | Yes |
| 7) Explain any patient exclusions from analysis | No | Yes |
| 8) Describe how confounding was assessed and/or controlled. | Yes | Yes |
| 9) If applicable, explain how missing data were handled in the analysis | Unclear | No |
| 10) Summarize patient response rates and completeness of data collection | Unclear | Unclear |
| 11) Clarify what follow-up, if any, was expected and the percentage of patients for which incomplete data or follow-up was obtained | Unclear | Unclear |
